# A MADS-box gene *NtSVP* regulates pedicel elongation by directly suppressing a *KNAT1*-like KNOX gene *NtBPL* in tobacco (*Nicotiana tabacum* L.)

**DOI:** 10.1093/jxb/erv332

**Published:** 2015-07-14

**Authors:** Di Wang, Xiaobo Chen, Zenglin Zhang, Danmei Liu, Gaoyuan Song, Xingchen Kong, Shuaifeng Geng, Jiayue Yang, Bingnan Wang, Liang Wu, Aili Li, Long Mao

**Affiliations:** ^1^National Key Facility for Crop Gene Resources and Genetic Improvement (NFCRI), MOA Key Laboratory of Crop Germplasm and Biotechnology, Institute of Crop Science, Chinese Academy of Agricultural Sciences (CAAS), Beijing 100081, China; ^2^Key Laboratory for Tobacco Gene Resources, Tobacco Research Institute, Chinese Academy of Agricultural Sciences, Qingdao 266101, China; ^3^School of Life Science, Shanxi University, Taiyuan 030006, China

**Keywords:** *BP*, gibberellin, MADS-box transcription factor, pedicel, *SVP*, tobacco.

## Abstract

Optimal inflorescence architecture is important for plant reproductive success by affecting the ultimate number of flowers that set fruits and for plant competitiveness when interacting with biotic or abiotic conditions. The pedicel is one of the key contributors to inflorescence architecture diversity. To date, knowledge about the molecular mechanisms of pedicel development is derived from *Arabidopsis*. Not much is known regarding other plants. Here, an SVP family MADS-box gene, *NtSVP*, in tobacco (*Nicotiana tabacum*) that is required for pedicel elongation was identified. It is shown that knockdown of *NtSVP* by RNA interference (RNAi) caused elongated pedicels, while overexpression resulted in compact inflorescences with much shortened pedicels. Moreover, an *Arabidopsis BREVIPEDECELLUS/KNAT1* homologue *NtBP-Like* (*NtBPL*) was significantly up-regulated in *NtSVP*-RNAi plants. Disruption of *NtBPL* decreased pedicel lengths and shortened cortex cells. Consistent with the presence of a CArG-box at the *NtBPL* promoter, the direct binding of NtSVP to the *NtBPL* promoter was demonstrated by yeast one-hybrid assay, electrophoretic mobility shift assay, and dual-luciferase assay, in which NtSVP may act as a repressor of *NtBPL*. Microarray analysis showed that down-regulation of *NtBPL* resulted in differential expression of genes associated with a number of hormone biogenesis and signalling genes such as those for auxin and gibberellin. These findings together suggest the function of a MADS-box transcription factor in plant pedicel development, probably via negative regulation of a *BP*-like class I KNOX gene. The present work thus postulates the conservation and divergence of the molecular regulatory pathways underlying the development of plant inflorescence architecture.

## Highlight

A tobacco (*Nicotiana tobaccum*) SVP family MADS-box gene is identified as a new regulator working in pedicel elongation for optimal inflorescence architecture by directly regulating an *Arabidopsis BP* homologue *NtBPL*

## Introduction

Inflorescence architecture plays critical roles in plant reproductive success by affecting the ultimate number of flowers that set fruits. Optimal inflorescence structure increases the competitiveness of plants when interacting with adverse environments (Evers *et al.*, 2011; Iwata *et al.*, 2012). Pedicel length is one of the key contributors to the structural diverstiy of plant inflorescences. In *Arabidopsis*, the class I KNOX gene *BREVIPEDICELLUS* (*BP*) was among the first to be found to affect pedicel development and inflorescence architecture (Venglat *et al.*, 2002). *BP* encodes the homeodomain protein KNAT1, a member of the *KNOX* (*KNOTTED1-LIKE HOMEODOMAIN*) family including *SHOOT MERISTEMLESS* (*STM*), *KNAT2*, and *KNAT6* that are involved in promoting stem cell division and delaying differentiation in the shoot apical meristem (SAM) (Hamant and Pautot, 2010; Hay and Tsiantis, 2010). Mutation of *BP* causes shortened pedicels and internodes because of fewer cell divisions. More profound defects were found for cell differentiation, elongation, and growth on the abaxial side than on the adaxial side of the pedicel, causing downward-pointing flowers and a compact inflorescence architecture, in addition to its pleitropic effect on the inflorescence stem and style.

*BP* is regulated by a number of genes. The phenotypes of the *BP* mutation become more severe in the mutants of *ERECTA* (*ER*) (van Zanten *et al.*, 2009), which may play a role redundant to *BP* in plant architecture regulation. ASYMMETRIC LEAVES 1 (AS1), a MYB transcription factor, acts in conjunction with AS2, a LATERAL ORGAN BOUNDARIES domain (LBD) transcription factor, to repress *BP* expression (Ori *et al.*, 2000; Guo *et al.*, 2008; Xu *et al.*, 2008) by recruiting components of the Polycomb-repressive complex2 to its promoter to generate an inactive chromatin state (Phelps-Durr *et al.*, 2005; Luo *et al.*, 2012; Lodha *et al.*, 2013). Recently, CINCINNATA-like TEOSINTE BRANCHED 1-CYCLOIDEA-PCF (TCP) transcription factors have been reported to interact with AS2 in the repression of class I *KNOX* genes (Li *et al.*, 2012). These studies suggest that KNOX repression may require different types of transcription factors in the development of different organs. Similar to their role in other plant organs, phytohormones such as auxins have been shown to be implicated in pedicel development. Mutation of *BIG*/*CRM1*, an auxin transport-related gene, causes shortened pedicels and internodes (Venglat *et al.*, 2002; Xu *et al.*, 2003; Douglas and Riggs, 2005; Yamaguchi *et al.*, 2007; Ragni *et al.*, 2008; Yamaguchi and Komeda, 2013). The severity of the corymb-like inflorescence in *crm1/big* mutants correlated with increased levels of *PIN1*, indicaitng that *CRM1*/*BIG* controls the elongation of the pedicels and stem internodes through auxin action (Yamaguchi and Komeda, 2013). Gain of function of the meristem identity regulator *LEAFY* (*LFY*) also produces downward-bending pedicels (Yamaguchi *et al.*, 2012). LFY can bind to the *AS2* promoter to enhance its activity directly, and subsequently suppresses *BP* expression (Xu *et al.*, 2003; Yamaguchi *et al.*, 2012).

MADS-box genes play important roles during plant development, especially floral organ development. The *SHORT VEGETATIVE PHASE* (*SVP*) MADS-box genes are of divergent functions in different plants. In *Arabidopsis*, the two SVP-clade genes *SVP* and *AGL24* have opposite functions in controlling the floral transition, with *AGL24* functioning as a promoter and *SVP* as a repressor (Hartmann *et al.*, 2000; Michaels *et al.*, 2003). In addition to this, *SVP* and *AGL24* act redundantly to control the identity of the floral meristem and to repress expression of class B, C, and E genes for regulating flower development (Gregis *et al.*, 2006, 2008, 2009). *JONTLESS* (*J*) is the tomato SVP homologue that is required for abscission zone (AZ) development (Mao *et al.*, 2000) and works with other MADS-box proteins SlMBP21 and MACROCALYX (MC) in the form of ternary protein complexes (Nakano *et al.*, 2012; Liu *et al.*, 2014). Mutation of *J* also causes an indeterminate inflorescence, suggesting its additional function to suppress the sympodial growth of tomato inflorescences (Szymkowiak and Irish, 2006). In snapdragon (*Antirrhinum majus*), the *SVP* homologue *INCOMPOSITA* (*INCO*) is responsible for prophyll development as well as floral meristem identity (Masiero *et al.*, 2004). Importantly, the involvement of *SVP*-like genes in determining inflorescence architecture was recently found to be conserved in *Arabidopsis* and rice (Liu *et al.*, 2013). Thus, study of the functions of SVP genes in additional species such as common tobacco (*Nicotiana tabacum* L.) would be interesting.

In this work, an *SVP*-like MADS-box gene *NtSVP* was identified in tobacco, another member of the Solanaceae family (Reinhardt and Kuhlemeier, 2002) in addition to tomato. It was found that, instead of working in flower AZ development and inflorescence determinacy like the tomato *SVP*-like gene *J*, *NtSVP* may play a major role in pedicel elongation. Moreover, NtSVP may directly regulate a downstream BP-like class I KNOX gene *NtBPL* as a transcription repressor. These findings should expand our understanding of the molecular mechanism that underlies plant inflorescence development.

## Materials and methods

### Plant material and growth conditions

Seeds of tobacco cv. W38 were obtained from the Tobacco Research Institute, Chinese Academy of Agricultural Sciences. All plants were grown at 24 °C in a greenhouse under long-day conditions (16h light/8h dark) with auxiliary light from sodium lamps.

### RACE and TAIL-PCR

THe *NtSVP* full-length cDNA sequence was amplified by the rapid amplification of cDNA ends (RACE) method. For 5’ RACE, primers *NtSVP*gstWR and Gene Racer 5’ primer were used. For 3’ RACE, primers AP, AUAP, *NtSVP*-F1, and *NtSVP*-F2 were used. The promoter sequence of *NtBPL* was amplified by thermal asymmetric interlaced PCR (TAIL-PCR) as previously described (Liu and Whittier, 1995) by AD primers (AD1–AD13) and three *NtBPL*-specific primers, *NtBPL*-SP1, *NtBPL*-SP2, and *NtBPL*-SP3, which were designed using expressed sequence tag (EST) sequences. All PCR constructs were subcloned into the pGEM-T Easy vector (Promega, USA), transformed into *Escherichia coil* DH5a cells, and sequenced. The primers used are shown in Supplementary Table S2 available at *JXB* online.

### Binary vector construction and tobacco transformation

RNA interference (RNAi) constructs were generated using pKANNIBAL (Wesley *et al.*, 2001) and pART27 vectors (Gleave, 1992). A 296bp region from the C-terminus of *NtSVP* and a 367bp region from the C-terminus of *NtBPL* were inserted into the pKANNIBAL vector twice in opposite directions with an intron between them to create a hairpin. The hairpin structure was then inserted into the binary vector pKART27. For construction of gene overexpression vectors, full-length open reading frames (ORFs) of *NtSVP* and *NtBPL* were inserted into pCHF3 binary vectors (Hajdukiewicz *et al.*, 1994) with gene-specific primers (Supplementary Table S2 at *JXB* online). After the sequences of the resulting constructs were confirmed, all vectors were transformed into the tobacco cv. W38 by *Agrobacterium tumefaciens*- (strain C58C1) mediated transformation using the leaf disc procedure (Horsch *et al.*, 1985) with timentin used as the bacteriostatic agent. Transgenic seeds were screened on half-strength Murashige and Skoog (MS) medium containing 100mg l^–1^ kanamycin.

### Quantitative RT-PCR (qRT-PCR)

Total RNA was extracted using an RNAprep pure plant kit (Tiangen, China) according to the manufacturer’s instructions. A 2 μg aliquot of total RNA was used for cDNA synthesis using TransScript One-Step gDNA Removal and cDNA Synthesis SuperMix (Transgen, China). qRT-PCR was performed on a ABI7300 qRT-PCR system (Applied Biosystems, USA) according to the manufacturer’s instructions using SYBR Premix Ex Taq™ II (Takara, Japan). The relative expression of each gene was calculated according to the 2^–ΔΔCt^ method (Livak and Schmittgen, 2001). All reactions were performed with three technical and three biological replicates. The data were normalized to the housekeeping gene *NtACTIN9*. The primers used are shown in Supplementary Table S2 at *JXB* online.

### Yeast one-hybrid assays

Bait plasmids were simultaneously heat transformed into yeast strain Golden yeast and selected on SD/–Ura agar medium. SMART technology synthesizes *NtSVP*-containing ends that are homologous to the end of the linearized pGADT7-Rec2 prey plasmid (Clontech). The linearized pGADT7-Rec vector and *NtSVP* in the bait-specific reporter strain were co-transformed into yeast cells and plated on aureobasidin A-containing selective medium according to the manufacturer’s instructions (Clontech, USA). The primers used are listed in Supplementary Table S2 at *JXB* online.

### Electrophoretic mobility shift assays (EMSAs)

The *NtSVP* full-length ORF was cloned into the pMAL-C2 vector (NEB, USA) using primer pair *Bam*HI-*NtSVP* Fw and *Xho*II-*NtSVP* Rv (Supplementary Table S2 at *JXB* online), and transformed into *E. coli* BL21 (DE3). Proteins were extracted from bacterial cells by ultrasonic crushing, and the cell lysate was purified by amylose resin affinity chromatography (NEB, USA) according to the manufacturer’s protocol. Probes containing the CArG-box and several flanking bases were labelled with biotin at the 3’ end. Unlabelled oligonucleotides of the same sequence were used as competitors. Oligonucleotides with a mutant CArG-box (AAATTATAAT) were used as negative control. A 1 μg aliquot of purified maltose-binding protein (MBP)–NtSVP protein and 50fmol biotin-labelled probes were used for the binding reaction for each sample. Florescence of biotin-labelled DNA was detected using the LightShift Chemiluminescent EMSA Kit (Pierce, USA). The oligonucleotides used are listed in Supplementary Table S2 at JXB online.

### Transient expression assay using a dual-luciferase system

The dual-luciferase assay was performed following a previously described method (Hellens *et al.*, 2005; Meng *et al.*, 2013). The effector plasmid 35S:*NtSVP* was constructed with the pCAMBIA2300 vector using the primer pair *Bam*HI-*NtSVP* Fw and *Xho*II-*NtSVP* Rv (Supplementary Table S2 at *JXB* online). The recombinant promoter containing the ~1kb native promoter of *NtBPL* amplified by the primer pair *Sal*I-pro*NtBPL*-Fw and *Bam*HI-pro*NtBPL*-RV and fused to the minimum 35S promoter was cloned into the vector pGreen-0800-LUC. Besides the wild-type CArG-box, two other reporter plasmids containing a mutant CArG-box in the *NtBPL* promoter were generated as controls using primers m1CArG-F, m1CArG-R, m2CArG-F, and m2CArG-R.

The reporter strain was incubated either alone or as a mixture with the effector strain (at the reporter:effector ratio of 1:1). The agrobacterial suspension in a 10ml syringe (without the metal needle) was carefully press-infiltrated onto healthy leaves of 1-month-old *N. benthamiana*. Plants were left in the dark for 1 d, and then moved to a greenhouse with long-day conditions (16h light/8h dark) for 2 d. Infiltrated leaf samples were sprayed with luciferin (1mM luciferin and 0.01% Triton X-100), and photographed. The luciferase activity was measured using the dual-luciferase reporter assay system (Promega, USA) according to the manufacturer’s instructions. The relative luciferase activity was calculated as the ratio between the firefly luciferase and the control *Renilla* luciferase activity. Five biological repeats were measured for each sample.

### Morphological analyses

To determine the mean pedicel lengths, pedicels were photographed at the anthesis stage, and measured using IMAGE J software. Values are means ±SE, based on 50 (*n*=10×5) pedicels from five individual plants per genotype. To determine the angles between pedicels and the inflorescence stem, 50 (*n*=10×5) pedicels from five plants were measured as a replication, and three replications were conducted.

For histological analysis, longitudinal sectioning was performed on pedicels fixed in FAA (3.7% formaldehyde, 5% acetic acid, 50% absolute ethanol). For cortex cell length and cortex cell number, 15 (*n*=3×5) pedicels at anthesis from five individual plants for each construct were sectioned longitudinally and photographed. To determine the mean pedicel cortex cell length, 30 cortex cells in a middle longitudinal cortex row were measured using IMAGE J software in each photograph. To determine the mean pedicel cortex cell number, all cortex cells at the middle sections were counted. This number was used to represent the total number of cortex cells in each pedicel. The number of cortex cells was counted in 15 sectioned pedicels for each genotype and averaged.

### Microarray analysis

Microarray assay was conducted by Shanghai Bio Corporation with two independent biological replicates, using an Agilent Whole Tobacco Genome Oligo Microarray (4×44K) (Agilent Technologies, Palo Alto, CA, USA). The chip hybridization results were scanned by the Agilent Microarray Scanner System and were normalized and analysed with Agilent Feature Extraction software. Differentially expressed genes with a significance threshold of *P*<0.05 and a >2-fold change between the *NtBPL*-RNAi transgenic plants and the wild type were selected. The description of each gene was annotated with *Arabidopsis* genes, which were obtained by a tblastx search against TAIR10 transcripts. Gene Ontology (GO) enrichment analysis was carried out by using proteins of *Arabidopsis* as templates at the AGRIGO website (http://bioinfo.cau.edu.cn/agriGO/). The *Arabidopsis* gene information was used for MapMan annotation (Thimm *et al.*, 2004)

## Results

### Identification of the *NtSVP* gene in tobacco

In light of the diverse functions of the *SVP* family genes, it was planned to explore the roles of *SVP*-like genes in tobacco. One homologue was cloned from *N. tabacum* cv. W38 using primers designed from the tomato *J* sequence and was named *NtSVP*. *NtSVP* is 1062bp in length with eight exons and seven introns, and encodes a 238 amino acid protein that shares ~77% similarity with the tomato J protein. It encodes a protein with four typical domains: a MADS-box domain, a variable I domain, a conserved K domain, and a C-terminal region (Supplementary Fig. S1A at *JXB* online). Phylogenetic analysis showed that *NtSVP* indeed belongs to the *SVP*-like lineage, with J and CaSVP as its closest homologues (Supplementary Fig. S1B available at *JXB* online). Interestingly, in contrast to most other members of the SVP-clade, *NtSVP* was highly expressed in pedicels and carpels in addition to vegetative organs such as leaf, stem, and root (Supplementary Fig. S1C available at *JXB* online).

### *NtSVP* regulates pedicel elongation

**Fig. 1.**
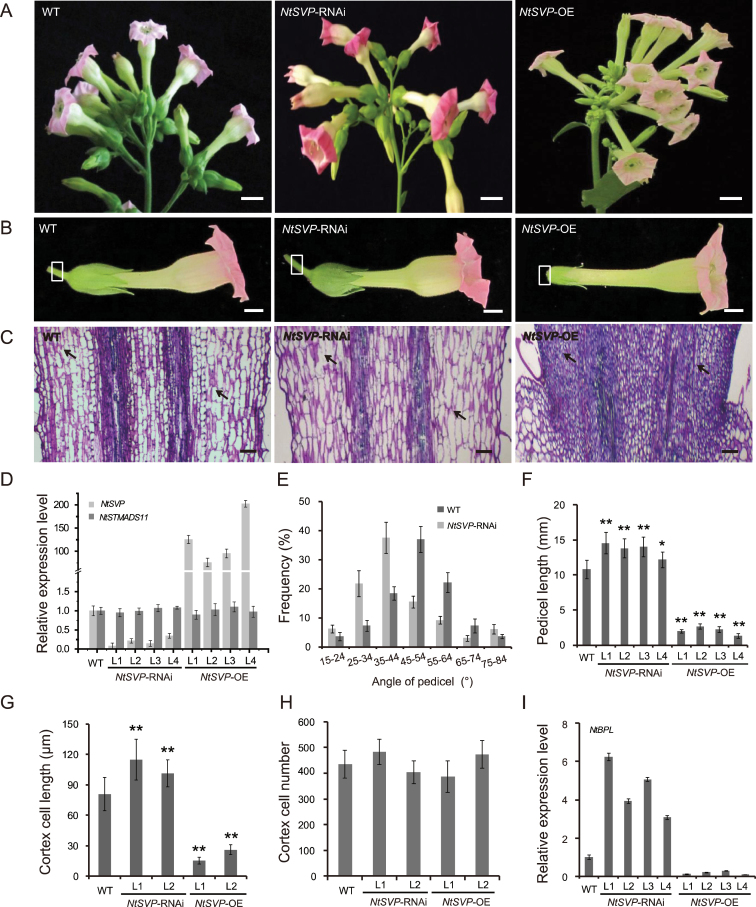
Characterization of *NtSVP* transgenic plants. (A) Inflorescence morphology of wild-type (WT), *NtSVP*-RNAi, and *NtSVP*-OE plants. Scale bars=20mm. (B) Representative flowers from WT, *NtSVP*-RNAi, and *NtSVP*-OE plants at anthesis. The portion of pedicel used for histological observation is indicated by a white box. Scale bars=10mm. (C) Longitudinal sections of the pedicels at anthesis of WT and *NtSVP* transgenic plants. Arrows point to cortex cell areas that are used for measurement. Scale bars=100 μm. (D) Detection of *NtSVP* and *NtSTMADS11* expression in *NtSVP* transgenic lines by qRT-PCR. The tobacco *NtACTIN9* gene was used as an internal control. (E) Distribution of the angles between the pedicels and the inflorescences in WT and *NtSVP*-RNAi plants. In total, 50 pedicels from five plants were measured as a replicate (*n*=10×5), and three replications were performed. Values are means ±SE. (F) Lengths of pedicels in WT, *NtSVP*-RNAi, and *NtSVP*-OE plants at anthesis (*n*=10×5, 50 pedicels from five individual plants per genotype). Asterisks indicate significant differences compared with the WT (*P<0.05, **P<0.01, Student’s *t*-test). (G) Quantification of pedicel cortex cell lengths of WT and *NtSVP* transgenic plants at anthesis (*n*=30×3×5, 15 pedicel sections from five individual plants per genotype were used and 30 cortex cells were measured for each section). Values are means ±SE. Asterisks indicate significant differences compared with the WT (**P<0.01, Student’s *t*-test). (H) Cell numbers in the longitudinal cortex file of pedicels in WT and *NtSVP* transgenic plants at anthesis (*n*=3×5, 15 sections from five individual plants per genotype were used). Values are means ±SE. (I) Detection of *NtBPL* expression in *NtSVP* transgenic lines by qRT-PCR. *NtACTIN9* was used as an internal control.

To characterize the function of *NtSVP*, RNAi transgenic lines were produced using a construct containing the less conserved C-terminal portion of *NtSVP* (Supplementary Fig. S2A at *JXB* online). Meanwhile, overexpression lines were generated using the DNA fragment containing the complete ORF. A total of 17 independent *NtSVP* RNAi lines (*NtSVP*-RNAi) and 11 independent *NtSVP* overexpression lines (*NtSVP*-OE) were obtained with evident inflorescence alteration when compared with that of the wild type (Fig. 1A–D). Unexpectedly, no obvious defect was observed in the pedicel AZ, suggesting functional divergence of *NtSVP* when compared with the tomato *SVP* homologue *J* (Supplementary Fig. S2B at *JXB* online).

Interestingly, RNAi lines had markedly stretched inflorescences when compared with those of wild-type plants (Fig. 1A). A closer observation revealed increased angles between the pedicel and the inflorescence. As shown in Fig. 1E, the range of pedicel angles in the wild-type plants was between 25 ° and 54 °, with a mean value of 42.8 °. In contrast, the average pedicel angle in RNAi lines was increased to 56.7 °, a 32.5% increase compared with the wild-type plants. Moreover, pedicels of RNAi lines were longer than those of the wild-type plants (Fig. 1B, F). The average length of pedicels in the RNAi transgenic lines was up to 13.7mm as measured from 50 flowers per phenotype, an increase of 26.2% when compared with the mean length of wild-type pedicels. Such elongation of pedicels persisted during flower development (Supplementary Fig. S2C, D at *JXB* online).

On the other hand, overexpression of *NtSVP* caused much shorter stature of the plants compared with the wild type (Supplementary Fig. S2E, F at *JXB* online). Remarkably, inflorescences became compacted due to significantly shortened pedicels (Fig. 1A, B). It was almost impossible to measure their lengths due to extremely small sizes. The average pedicel length for *NtSVP*-OE transgenic plants was ~2mm at anthesis (Fig. 1F), much shorter than those of the wild type (10.8mm). A longitudinal section of the pedicels showed altered elongated cortex cells at the pedicels of *NtSVP*-RNAi transgenic plants, while cortex cells of the overexpression lines were small and densely organized (Fig. 1C). To distinguish whether the change of pedicel length is caused by increased cell elongation or promoted cell division, cell lengths were measured at the cortical regions. The results showed that cortex cells were significantly elongated in RNAi lines. As shown in Fig. 1G, the average cortex cell length increased to >100 μm on average, when compared with 80 μm in wild-type pedicel cortex cells. On the other hand, cortex cells in *NtSVP* overexpression plants were extremely short, only 18–28% of the wild-type mean. Despite this, no significant difference was found for cell numbers in the transgenic plants and the wild-type control (Fig. 1H). These data suggest that *NtSVP* regulates pedicel development by affecting cell elongation, rather than cell proliferation. To test the possible off-target effect of RNAi, the correlation between expression levels of transgenes and pedicel lengths was studied. As shown in Fig. 1D, qRT-PCR assay showed that *NtSVP* transcript levels were indeed proportional to pedicel lengths in the transgenic plants (Fig. 1F), while another homologous MADS-box gene *NtSTMADS11* was not affected. These results demonstrate that the effect on pedicel elongation was indeed caused by altered expression of *NtSVP*.

### *NtSVP* suppresses the expression of *NtBPL*, a tobacco BP/KNAT1 homologue

In *Arabidopsis*, *BP* has been known as the major player in pedicel development (Venglat *et al.*, 2002; Douglas and Riggs, 2005). To study the functions of *BP* homologues in tobacco, the tobacco EST sequences were searched using *Arabidopsis BP* as a query. One EST (AF544053) showed high similarity to *Arabidopsis BP* and was named *NtBPL* (*Nicotiana tabacum BP-Like*). Full-length cDNA was then obtained using 5’ and 3’ RACE experiments. NtBPL protein contained four domains typically found in class I KNOX proteins, such as KNOX I and KNOX II domains (Supplementary Fig. S3A at *JXB* online). It shares 55.7% sequence similarity to the *Arabidopsis* BP and is closely related to thetomato class I KNOX gene *TKN1* (Supplementary Fig. S3B at *JXB* online). Similar to *Arabidopsis BP*, *NtBPL* was found to have the highest expression in tobacco pedicels (Supplementary Fig. S3C at *JXB* online). The *NtBPL* expression level in pedicels of *NtSVP* transgenic plants was determined and it was found that *NtBPL* was up-regulated in *NtSVP*-RNAi lines, and significantly suppressed when *NtSVP* was overexpressed (Fig. 1I). Furthermore, the expression level of *NtBPL* increased along with pedicel development stages, when the expression of *NtSVP* decreased from stage 1 to stage 4, indicating possible co-ordinated expression between *NtSVP* and *NtBPL* during tobacco pedicel development (Supplementary Fig. S4 at *JXB* online).

### *NtBPL* promotes pedicel elongation in tobacco

**Fig. 2.**
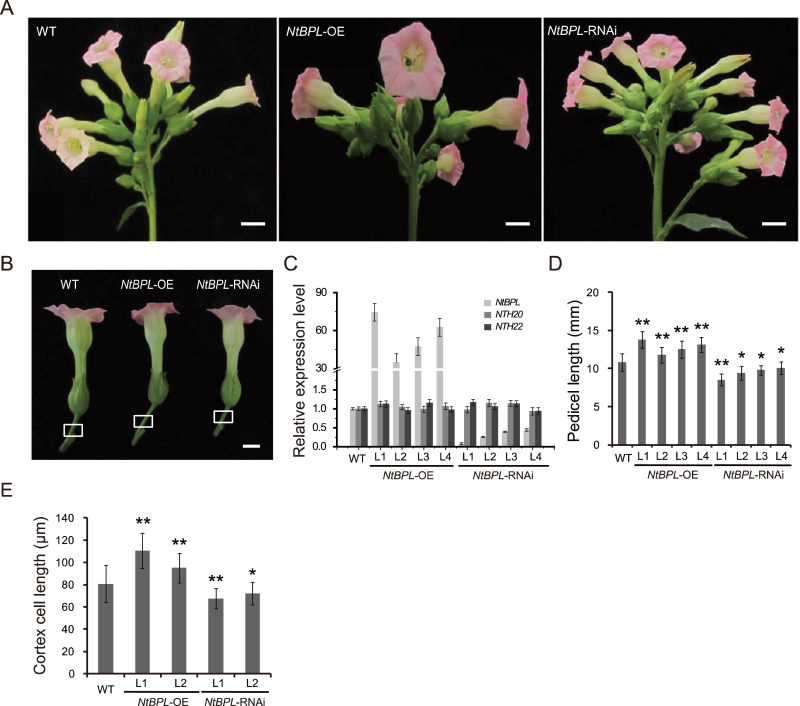
Characterization of *NtBPL* transgenic plants. (A) Inflorescence morphology of wild-type (WT), *NtBPL*-OE, and *NtBPL*-RNAi plants. Scale bar=20mm. (B) The flowers of WT, *NtBPL*-OE, and *NtBPL*-RNAi, with pedicels of different lengths as indicated by white boxes. Scale bars=10mm. (C) Detection of the expression levels of *NtBPL* and two closely related homologues *NTH20* and *NTH22* in *NtBPL* transgenic lines by qRT-PCR. *NtACTIN9* was used as an internal control. (D) Lengths of pedicels in WT, *NtBPL*-OE, and *NtBPL*-RNAi plants at anthesis (*n*=8×5, 40 pedicels from five individual plants per genotype). Values are means ±SE. Asterisks indicate significant differences compared with the WT (*P<0.05, **P<0.01, Student’s *t*-test). (E) Quantification of pedicel cortex cell lengths of WT and *NtBPL* transgenic plants at anthesis (*n*=30×3×5, 15 pedicel photographs were prepared from five individual plants, and 30 cortex cells were measured for each photograph). Values are means ±SE. Asterisks indicate significant differences compared with the WT (*P<0.05, **P<0.01, Student’s *t* test).

To identify whether *NtBPL* plays a role in tobacco pedicel development, transgenic plants were generated comprising 18 *NtBPL*-RNAi lines and 15 *NtBPL*-OE lines. Four lines for each genotype were further studied; these exhibited clear morphological changes in their inflorescences compared with wild-type plant (Fig. 2A, C). As shown in Fig. 2C, the expression of *NtBPL* was down-regulated in RNAi lines and increased in OE lines, while two closely related genes *NTH20* and *NTH22* were not affected. Unlike *NtSVP* transgenic plants, transgenic manipulation of *NtBPL* did not affect tobacco plant height (Supplementary Fig. S5A at *JXB* online), nor did this have a clear effect on pedicel angles (Supplementary Fig. S5B at *JXB* online). Despite this, knockdown of *NtBPL* reduced pedicel lengths an average of 1.8mm, or 16% of that of the wild type, while overexpression of *NtBPL* caused a significant increase in pedicel length, up to 13.8mm, or a 27.8% increase, as measured from 50 flowers per phenotype (Fig. 2B, D). These data indicate that *NtBPL* is closely correlated with pedicel elongation and indeed required for pedicel development. Accordingly, cortex cells were found to be reduced ~10% in *NtBPL*-RNAi plants and increased >15% when *NtBPL* was overexpressed (Fig. 2E; Supplementary Fig. S5C at *JXB* online). Because the total number of cortex cells remained statistically unchanged, the variation of pedicel lengths can be attributed to the elongation of cortex cells (Supplementary Fig. S5D at *JXB* online).

**Fig. 3.**
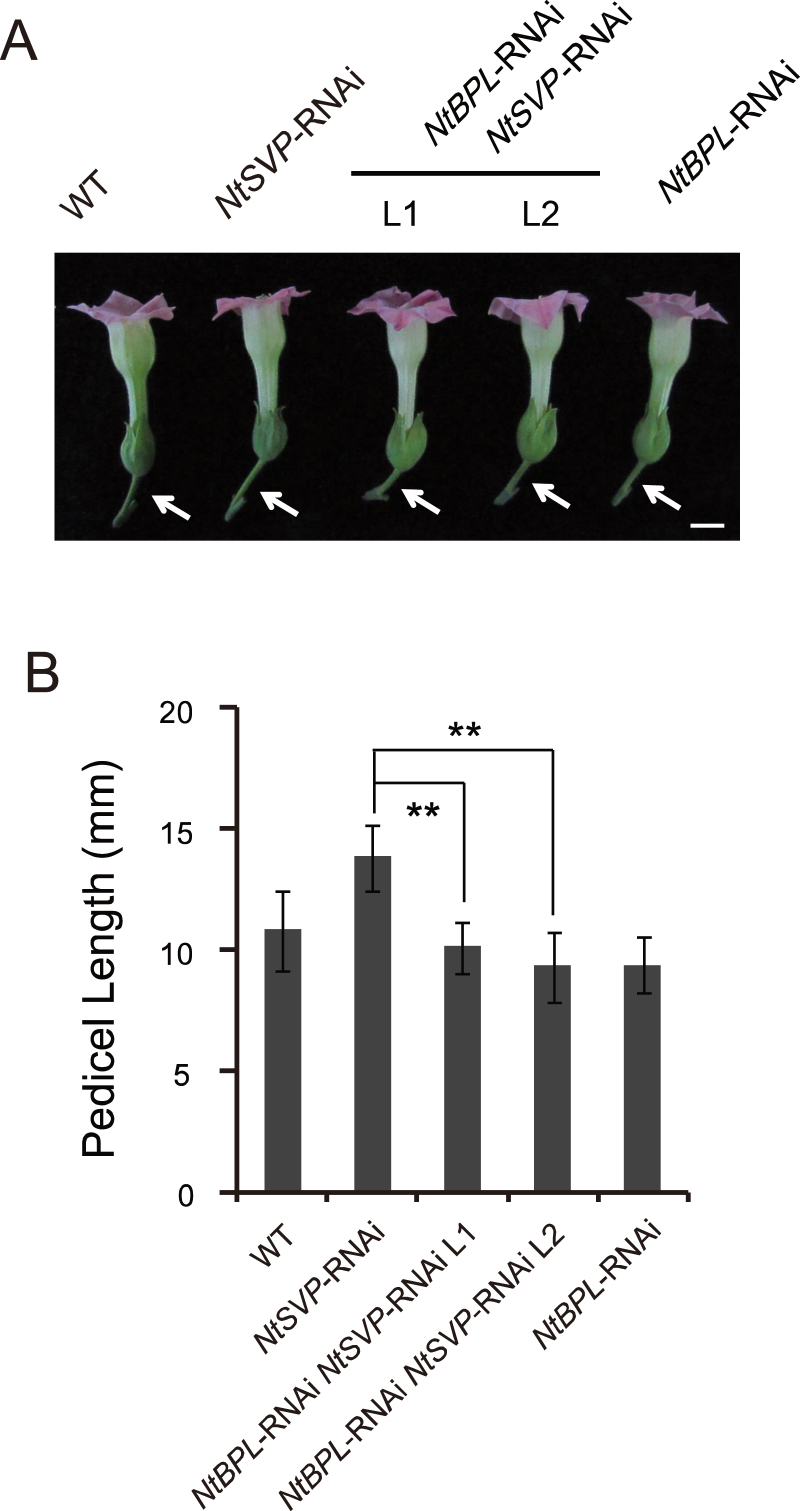
Genetic interaction of *NtSVP* and *NtBPL* by double RNAi analysis. (A) Pedicels of flowers at anthesis from wild-type (WT), *NtSVP*-RNAi, *NtBPL NtSVP* double RNAi plants L1 and L2, and *NtBPL*-RNAi plants. Scale bar=10mm (B) Quantitative measurement of pedicel lengths of the plants described in (A). Values are means ±SE (*n*=8×5, 40 pedicels from five individual plants per genotype). **P<0.01 by Student’s *t*-test.

The expression level of *NtSVP* was then checked in pedicels of *NtBPL* transgenic plants, and no significant change was found (Supplementary Fig. S5E at *JXB* online), indicating that *NtBPL* is indeed located downstream of *NtSVP* and there was no feedback regulation on *NtSVP*. To confirm this notion further, *NtSVP/NtBPL* double RNAi plants were generated by crossing RNAi lines of the two genes. The *NtSVP* and *NtBPL* double RNAi plants exhibited shortened pedicels, similar to those of *NtBPL*-RNAi plants, suggesting a downstream location of *NtBPL* relative to *NtSVP* (Fig. 3). In other words, *NtSVP* and *NtBPL* may possibly work on the same regulatory pathway for tobacco pedicel development.

### *NtSVP* acts as a transcription repressor of *NtBPL* by directly binding to its promoter

**Fig. 4.**
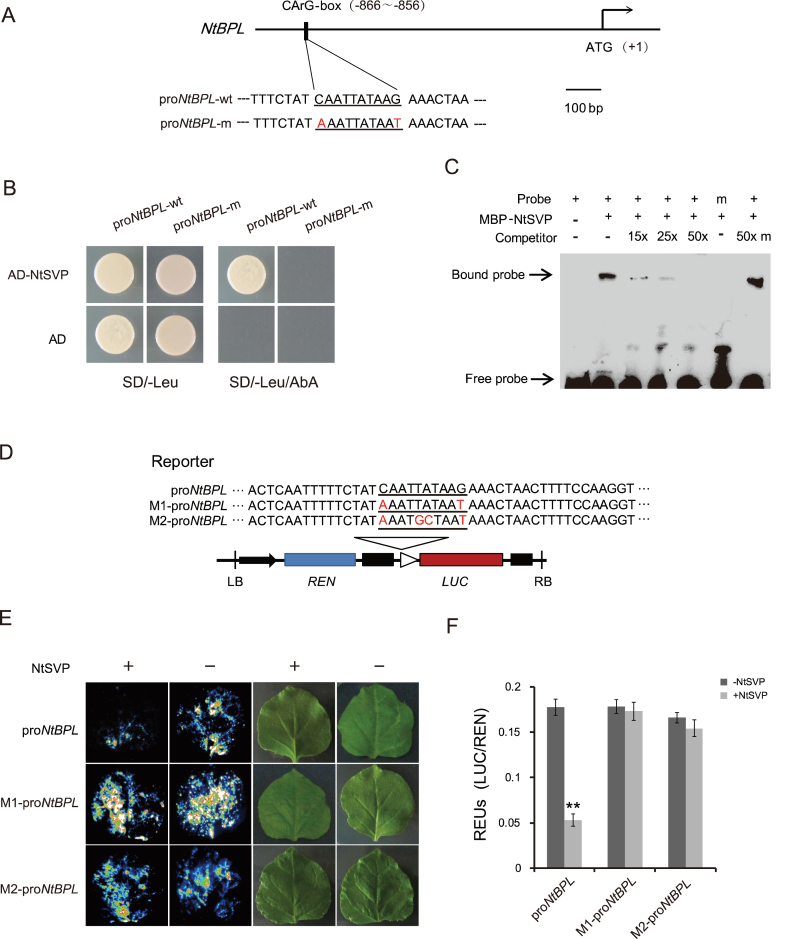
NtSVP as a direct transcription repressor of *NtBPL*. (A) The design of the wild-type (WT) probe containing the CArG-box (pro*NtBPL*-wt) and the mutated probe (pro*NtBPL*-m) in the *NtBPL* promoter. Mutated bases are highlighted in red. Numbers indicate sequence locations. (B) Yeast one-hybrid assays showing interactions between the NtSVP protein and the *NtBPL* promoter. pGADT7 (AD) was used as control. (C) EMSA showing MBP–NtSVP fusion protein binding to the probes of pro*NtBPL* as described in (A). m represents pro*NtBPL*-m as a control. + indicates the presence and – the absence of corresponding components as indicated. (D) The design of reporter constructs for the dual-luciferase assay. The WT and two mutant version of pro*NtBPL* (M1, M2) are indicated. (E) LUC activities in *N. benthamiana* leaves infiltrated with the *Agrobacterium* strain harbouring the indicated reporter in the presence (+) or absence (–) of the co-transfected *Agrobacterium* strain harbouring the plasmid expressing the NtSVP protein. (F) Dual-luciferase assay of relative reporter activity of samples shown in (E). The relative LUC activities were normalized to REN activity and presented as relative expression units (REUs, *n*=5). **P<0.01.

Since genetic analysis indicated that *NtBPL* acted downstream of *NtSVP*, experiments were carried out to determine whether *NtBPL* was a direct target of *NtSVP* regulation. To test this hypothesis, ~1kb of the promoter sequence of *NtBPL* was cloned and *cis*-element prediction was performed using the PLACE program. A CArG-box (5’CAATATTAAG3’), a consensus MADS-box transcription factor binding site, was found on the *NtBPL* promoter at –866bp to –856bp from the initiation codon (Fig. 4A; Supplementary Fig. S6 at *JXB* online), providing a condition for direct binding of NtSVP to the promoter of *NtBPL*. Yeast one-hybrid assay showed that NtSVP could indeed activate the expression of a reporter gene driven by the *NtBPL* promoter (Fig. 4B). This was further confirmed by EMSA using the MBP–NtSVP fusion protein. As shown in Fig. 4C, the MBP–NtSVP fusion protein was able to bind to DNA probes containing the wild-type CArG-box, but failed to bind to the mutated version. Furthermore, increasing the concentration of unlabelled wild-type probes in the binding reactions resulted in much weaker retarded bands. Meanwhile, the formation of pro*NtBPL* and the MBP–NtSVP complex was not inhibited by an excess amount of mutated probes, indicating that NtSVP may specifically bind to the *NtBPL* promoter under these conditions.

Next a transient expression assay was performed using the dual-luciferase system in *N. benthamiana* leaves to study further the mode of action of *NtSVP* on the transcription of *NtBPL*. Three reporter constructs were generated, one containing the wild-type CArG-box and the other two containing mutated CArG-boxes (Fig. 4D). An effector plasmid expressing the full-length NtSVP protein was also constructed. As shown in Fig. 4E, when the NtSVP effector was co-expressed with the LUC reporter gene driven by the *NtBPL* promoter, the expression of LUC reporter genes was significantly repressed. On the other hand, when the reporter construct carried a mutated CArG-box, no significant change in LUC activity was observed whether NtSVP was present or not (Fig. 4E, F). These results support the idea that *NtSVP* is a repressor of *NtBPL* and negatively regulates its expression by direct binding to its promoter.

### Global gene expression analysis indicates involvement of hormonal regulation in tobacco pedicel development

To investigate further genes that are involved in tobacco pedicel development, the transcriptomes in the pedicels of wild-type and *NtBPL*-RNAi plants were compared using Agilent tobacco microarrays (http://sbc.biomart.cn). A total of 657 differentially expressed genes were detected between the wild-type and RNAi pedicels [absolute log2 value ≥1; *P*-value ≤0.001; false discovery rate (FDR) ≤0.01] (Supplementary Dataset 1 at *JXB* online). Among them, 427 were down-regulated and 230 were up-regulated when *NtBPL* was knocked down. Four genes were randomly selected for qRT-PCR detection, and the results confirmed the consistency of the microarray data (Supplementary Fig. S7 at *JXB* online). GO enrichment analysis showed that genes for response to stimulus and various abiotic stresses were significantly enriched among those down-regulated genes (Supplementary Fig. S8A at *JXB* online). There were fewer enriched classes of genes among up-regulated genes, including ‘response to stimulus’, ‘localization’, ‘transport’, and ‘cellular amino acid and derivative metabolic process’ functions (Supplementary Fig. S8B at *JXB* online).

**Fig. 5.**
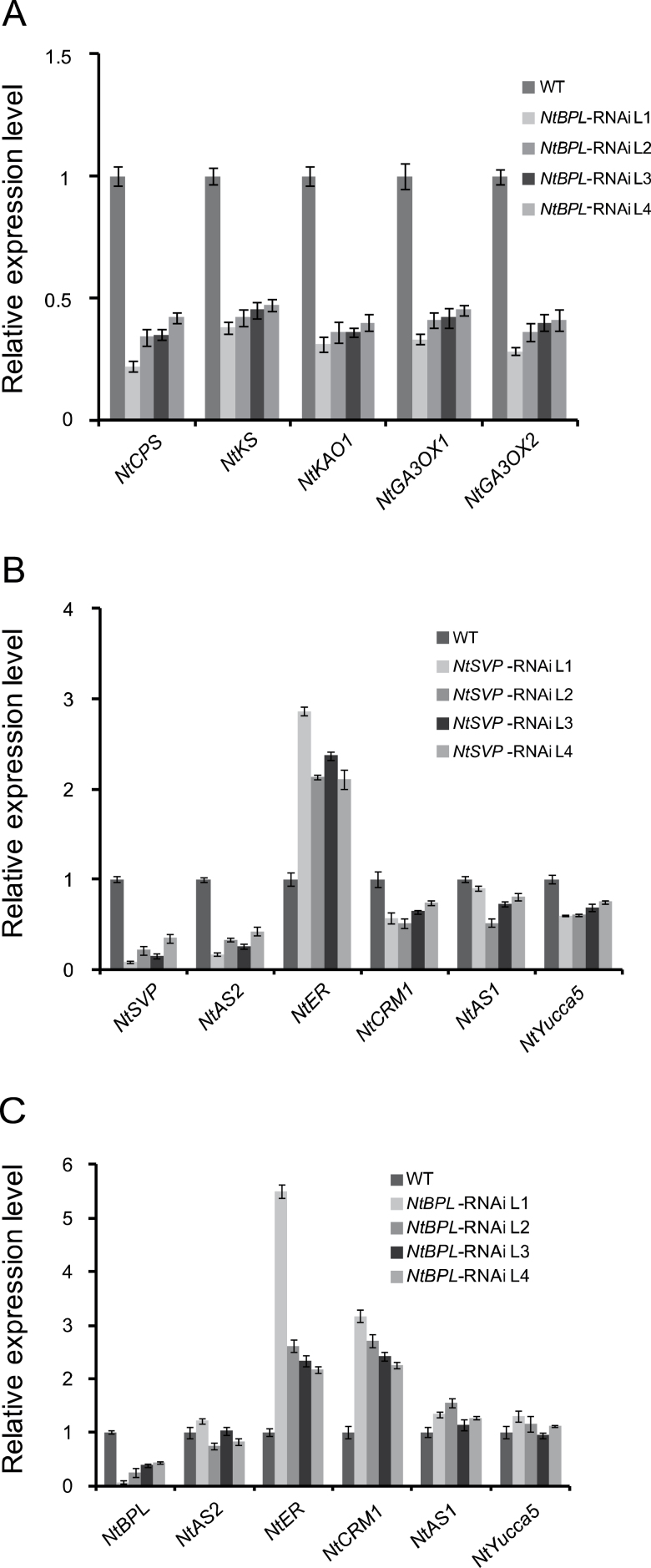
Expression analysis of tobacco homologues of *Arabidopsis* genes for pedicel development. (A) qRT-PCR detection of transcript levels of differently expressed GA biosynthetic genes (Heinrich *et al*., 2013) in pedicels of *NtBPL*-RNAi plants compared with the wild type (WT) according to microarray results. *NtACTIN9* was used as an internal control. (B) qRT-PCR detection of transcript levels of homologues to *Arabidopsis* pedicel-regulating genes in pedicels of *NtSVP*-RNAi and WT plants. Sequence information was obtained from the SOL Genomics Network by Blast using *Arabidopsis* genes as queries. *NtAS1*, mRNA_73569; *NtAS2*, mRNA_104917; *NtER*, mRNA_128857; *NtCRM1*, mRNA_59455; *NtYucca5*, mRNA_8257). (C) qRT-PCR detection of transcript levels of the above genes in pedicels of *NtBPL*-RNAi and WT plants.

Those differentially expressed genes were then further classified using the MapMan program (http://mapman.gabipd.org), and it was found that the hormone metabolism pathway was notably enriched among down-regulated genes (Supplementary Fig. S8C at *JXB* online). Hormone biogenesis and signalling genes for auxin, ethylene, gibberellin (GA), and brassinosteriod were found among the differentially expressed genes (Supplementary Table S1 at *JXB* online). There were seven genes associated with GA functions, five of which were for GA biosynthesis, including homologues of *ent-copalyl diphosphate synthase* (*CPS*), *ent-kaurene synthase* (*KS*), *ent-kaurenoic acid oxidase* (*KAO*), and *GA 3-oxidase* (*GA3OX1/2*) (Yamaguchi, 2008). These genes were subject to further characterization by qRT-PCR. As shown in Fig. 5A, *NtCPS*, *NtKS*, and *NtKAO1* were down-regulated in *NtBPL*-RNAi lines, together with two homologues, *NtGA3OX1* and *NtGA3OX2*, whose products putatively catalyse the last step of GA biogenesis. These data indicate that disruption of *NtBPL* may significantly affect GA biogenesis which may play a significant role in tobacco pedicel development. This is consistent with GA as a key hormone for plant cell elongation and division (Shani *et al.*, 2013).

In *Arabidopsis*, genes that are known to be involved in pedicel development include *AS1/2*, *ER*, *CRM1*, and *Yucca5*. To test the possible relationships of these genes in the *NtSVP–NtBPL* pathway, homologues of these genes were isolated from tobacco and their expression patterns were studied in *NtSVP* and *NtBPL*-RNAi plants. As shown in Fig. 5B and C, *NtAS2* was significantly down-regulated in *NtSVP*-RNAi plants. *NtER*, however, was up-regulated when either *NtSVP* or *NtBPL* was down-regulated. It is not clear how these two genes were integrated into the *NtSVP–NtBPL* pathway in tobacco. In addition, the tobacco *CRM1* homologue *NtCRM1* was significantly up-regulated in *NtBPL*-RNAi lines. These data suggest that auxin and perhaps additional hormones are involved in tobacco pedicel development as has been shown in *Arabidopsis* (Yamaguchi and Komeda, 2013). Thus the present work put forward an improved regulatory model for plant pedicel development.

## Discussion

### Functional divergence of the *SVP* family genes

MADS-box genes are ubiquitous among flowering plants. Many floral development-associated MADS-box genes are conserved in distantly related species, although subfunctionalization has been reported for genes with duplicated copies (Rijpkema *et al.*, 2006). Despite this, existing data show that genes in the *SVP* family are divergent in functions even in closely related species. The tomato J and its interacting partners MACROCALXY (MC) and SEPALLATA-like SlMBP21 are essential for AZ formation in tomato (Mao *et al.*, 2000; Nakano *et al.*, 2012; Liu *et al.*, 2014). *J* is also involved in determining the sympodial meristem identity and hence the tomato inflorescence architecture (Szymkowiak and Irish, 2006). In contrast, the *Arabidopsis SVP* works as a flowering repressor (Hartmann *et al.*, 2000), while a second SVP-like MADS-box gene *AGL24* is a flowering promoter (Michaels *et al.*, 2003). In snapdragon, *INCO* is responsible for prophyll development as well as floral meristem identity (Masiero *et al.*, 2004). In woody perennial vine kiwifruit (*Actinidia* spp.), overexpression of an *SVP* gene affects reproductive development and suppresses anthocyanin biosynthesis in petals, but has no effect on vegetative growth, dormancy, or flowering time (Wu *et al.*, 2014). The involvement of *SVP* or *SVP*-like genes in genetic pathways determining inflorescence architecture was recently found to be conserved in *Arabidopsis* and rice (Liu *et al.*, 2013). Other spike-related phenotypes in grass species may also be related to pedicel development such as those caused by rice *Dense and Erect Panicle1* (*DEP1*), *DEP2*, and *DEP3* (Huang *et al.*, 2009; Li *et al.*, 2010; Qiao *et al.*, 2011; Jiang *et al.*, 2014). Although common tobacco belongs to the Solanaceae family, functional divergence of the *SVP* family genes may also occur. This is in line with the findings that *NtSVP* is involved in pedicel development and does not seem to be implicated in AZ development, in contrast toclosest tomato homologue *J* (Mao *et al.*, 2000). Such an observation may contribute to the possibility of the existence of additional homologues of *SVP* in tobacco.

### Conservation and divergence of regulatory pathways for plant pedicel development

*KNOX* genes regulate various aspects of development in the plant kingdom and play a key role in controlling the establishment and maintenance of the SAM (Hay and Tsiantis, 2009, 2010). In *Arabidopsis*, the class I *KNOX* gene *BP* is the major player in pedicel development. In tobacco, *NtBPL* has conserved, but not identical, functions. *NtBPL* is involved in pedicel elongation; its effect on the pedicel angle seems to be limited. The average pedicel angles are widened, but not as severely as in an *Arabidopsis bp* mutant where flowers became downward pointing (Venglat *et al.*, 2002). In *Arabidopsis*, *BP* is a repressor of *KNAT6* and the *ARABIDOPSIS THALIANA HOMEOBOX GENE1* (*ATH1*) gene, which was not observed among differentially expressed genes in the present microarray data. One possibility is that the molecular pathways for pedicel development may be different between the two species. The other possibility is that the corresponding gene is not included in the current version of the tobacco microarray. Also no regulatory relationship between AS1/2 homologues and NtBPL was observed, while in *Arabidopsis*, the AS1–AS2 complex represses *BP* and *KNAT2* (Phelps-Durr *et al.*, 2005; Luo *et al.*, 2012; Lodha *et al.*, 2013). On the other hand, no MADS-box transcription factor has been shown to be involved in pedicel development. Thus, the discovery of an *SVP*-like MADS-box gene as a repressor for *NtBPL* in tobacco may represent a divergent, or as yet uncovered regulatory step for plant pedicel development.

### An extended molecular pathway for tobacco pedicel development

In *Arabidopsis*, a number of genes have been reported to regulate *BP* in pedicel development. In addition to *AS1* and *AS2* as repressors of *BP* and *KNAT2* (Lin *et al.*, 2003; Xu *et al.*, 2003; Guo *et al.*, 2008; Rast and Simon, 2012), *TCP* transcription factors have recently been reported to interact with AS2 to repress class I *KNOX* genes (Li *et al.*, 2012). Thus, the specification of pedicel development is strictly regulated, with the implication of a variety of transcription factors, mostly playing roles as suppressors. Such a mode of action seems to be conserved in tobacco. Here, *NtSVP*, a MADS-box transcription factor gene, is found to be associated with tobacco pedicel development as a transcription suppressor of *NtBPL*. The data suggest that NtSVP may exert its function on pedicel development by direct binding to the promoter of *NtBPL*. Interestingly, *NtAS2* was found to be severely suppressed in *NtSVP*-RNAi pedicels, putting *NtAS2* in a position in between *NtSVP* and *NtBPL*. The molecular mechanism of how NtSVP interacts with other *NtBPL* regulators such as *AS1/2* and *TCP* genes as shown in *Arabidopsis* needs further investigation.

### Hormonal regulation of tobacco pedicel development

In *Arabidopsis*, auxin is considered to be involved in pedicel development, as shown by the mutation of the auxin transport-related gene *BIG*/*CRM1* that results in shortened pedicels and internodes (Douglas and Riggs, 2005; Yamaguchi and Komeda, 2013). The function of *CRM1* is positively correlated with the activity of the auxin transporter *PIN1*. However, *BP* affects several aspects of pedicel development, including cell division, cell differentiation, and cell elongation, with no polarity in either internode or pedicel, indicating that as far as *BP* is concerned, auxin may not be the major downstream hormonal effector (Venglat *et al.*, 2002).

Transcriptome analysis showed that when *NtBPL* was disrupted, several classes of hormones were affected; however, genes involved in GA biosynthesis and signalling were more likely to be responsible for pedicel development in tobacco. The functions of KNOX proteins in regulating GA-related genes (*GA20OX* and *GA2OX*) have been reported in *Arabidopsis* stems and shoot meristem, where KNOX proteins induce cytokinin biosynthesis, directly suppress GA synthesis via GA 20-oxidase repression, and promote GA deactivation via GA 2-oxidase activation (Sakamoto *et al.*, 2001; Hay *et al.*, 2002). Furthermore, in the triple mutants of the gibberellin receptor genes *GID1a-1*, *GID1b-1*, and *GID1c-1*, the elongation of the pedicel is strikingly reduced when compared with the single mutant (Griffiths *et al.*, 2006). Transcriptome analysis revealed seven GA-related genes that were differentially expressed when *NtBPL* was disrupted. Such an observation supports the idea that GA may be the major player for tobacco pedicel development. Such a hypothesis should be studied in more detail.

Tobacco has been a research model for a long time despite its tetraploid nature. The high similarity between the homoeologues makes it feasible to carry out functional characterization by manipulating the copies simultaneously. Nevertheless, a number of questions related to *NtSVP* and *NtBPL* genes remain to be answered. Would additional copies of the *NtSVP* gene be responsible for AZ development as its closest homologue is in tomato? Since *AS2* seems to be down-regulated in *NtSVP*-RNAi lines and AS2 is known to suppress *BP* in *Arabidopsis*, how does *NtSVP* work together with *AS2* in regulating *NtBPL*? What kind of genes are the direct targets of *NtBPL*? Future study using tobacco as a model should provide interesting insight into the molecular mechanisms to fine-tune plant pedicel development.

## Supplementary data

Supplementary data are available at *JXB* online.

Figure S1. Protein sequence analysis and tissue-specific expression of *NtSVP*.

Figure S2. Phenotypes of the *NtSVP* transgenic plants.

Figure S3. Protein sequence analysis and tissue-specific expression of *NtBPL*.

Figure S4. Developmental-specific expression of *NtSVP* and *NtBPL* in pedicels of wild-type plants.

Figure S5. Phenotypes of the *NtBPL* transgenic plants.

Figure S6. The promoter sequence of *NtBPL.*

Figure S7. Confirmation of microarray data.

Figure S8. GO enrichment analysis of differentially expressed genes in *NtBPL*-RNAi pedicels and their MapMan classification.

Table S1. Hormone-related genes that are differentially expressed due to down-regulation of *NtBPL*.

Table S2. Primers used in this work.

Dataset 1. List of differentially expressed genes between the wild-type and *NtBPL*-RNAi pedicels.

Supplementary Data
