# Supplementary material for: A MADS-box gene NtSVP regulates pedicel elongation by directly suppressing a KNAT1-like KNOX gene NtBPL in tobacco (Nicotiana tabacum L.)
Source: J Exp Bot. 2015 Jul 14;66(20):6233–44. doi: 10.1093/jxb/erv332 (PMC4588881; doi:10.1093/jxb/erv332)
Supplement: Supplementary Data [file supp_66_20_6233__index.html]

A MADS-box gene NtSVP regulates pedicel elongation by directly suppressing a KNAT1-like KNOX gene NtBPL in tobacco (Nicotiana tabacum L.) — A MADS-box gene NtSVP regulates pedicel elongation by directly suppressing a KNAT1-like KNOX gene NtBPL in tobacco (Nicotiana tabacum L.) — Supplementary Data 

# A MADS-box gene *NtSVP* regulates pedicel elongation by directly suppressing a *KNAT1*-like KNOX gene *NtBPL* in tobacco (*Nicotiana tabacum* L.)

## Supplementary Data

Data files

- Supplementary Data - Supplementary Data
- Supplementary Data - Supplementary Data
