# Supplementary material for: A MADS-box gene NtSVP regulates pedicel elongation by directly suppressing a KNAT1-like KNOX gene NtBPL in tobacco (Nicotiana tabacum L.)
Source: J Exp Bot. 2015 Jul 14;66(20):6233–44. doi: 10.1093/jxb/erv332 (PMC4588881; doi:10.1093/jxb/erv332)
Supplement: Supplementary Data [file supp_erv332_jexbot151217_file001.pdf]

A MADS-box gene *NtSVP* regulates pedicel elongation by directly suppressing a *KNAT1*-like KNOX gene *NtBPL* in tobacco (*Nicotiana tobacum* L.)

Di Wang[1,*], Xiaobo Chen[1,*], Zenglin Zhang[2], Danmei Liu[3], Gaoyuan Song[1], Xingchen Kong[1], Shuaifeng Geng[1], Jiayue Yang[1], Bingnan Wang[1], Liang Wu[1], Aili Li[1], and Long Mao[1,]**

[1] National Key Facility for Crop Gene Resources and Genetic Improvement (NFCRI), MOA Key Laboratory of Crop Germplasm and Biotechnology, Institute of Crop Science, Chinese Academy of Agricultural Sciences (CAAS), Beijing 100081, China

[2] Key Laboratory for Tobacco Gene Resources, Tobacco Research Institute, Chinese Academy of Agricultural Sciences, Qingdao 266101, China

[3] School of Life Science, Shanxi University, Taiyuan 030006, China

*These authors contributed equally to this work.

**For correspondence: maolong@caas.cn

Supplementary figure legends

Supplementary Fig. S1. Protein sequence analysis and tissue-specific expression of *NtSVP*.

(A) Alignment of SVP-like protein sequences . The MADS-box, I domain, K domain and C-terminal domain are marked. Amino acids in black shading and grey shading indicate identical or similar amino acids among the proteins.

(B) An unrooted phylogenetic tree of SVP-clade MADS-box proteins from some species. The scale indicates branch lengths. Accession numbers are as follows: OsMADS22 (Os02g0761000), OsMADS47 (Os03g0186600), OsMADS55 (Os06g0217300), HvBM1 (AJ249142), HvBM10 (EF043040), Brassica campestris (Chinese cabbage) BcSVP (DQ922945), and CaSVP (AFI49342), Eucalyptus grandis EgrSVP (AY263809), NtSTMADS11 (mRNA_51421 in SOL Genomics Network), INCO (CAG27846), JOINTLESS (Solyc11g010570), SVP (At2g22540), AGL24 (At4g24540), Ta-VRT2(DQ022679), SOC1 (At2g45660).

(C) Tissue-specific expression of *NtSVP* in wild-type plants.

Supplementary Fig. S2. Phenotypes of the *NtSVP* transgenic plants.

(A) DNA fragment used for *NtSVP*-RNAi construct.

(B) The morphology of abscission zones in wild-type and *NtSVP*-RNAi transgenic tobacco indicated by white arrows.

(C) The morphology of flower and pedicel in the five developmental stages as described (Zhu *et al*., 2013).

(D) Quantitative measurement of the pedicel lengths of the WT and two *NtSVP*-RNAi lines from S1 to S5 (N = 10 X 5, 50 pedicels from five individual plants per genotype). Values are means ± SE.

(E) The whole plant morphology of wild-type and *NtSVP* transgenic plants at the reproductive stage.

(F) Quantitative measurement of plant height of WT, *NtSVP*-RNAi and *NtSVP*-OE plants at anthesis stage. (N=5, 5 plants for each genotype). Values are means ± SE.

Supplementary Fig. S3. Protein sequence analysis and tissue-specific expression of *NtBPL*.

(A) Alignment of the NtBPL protein sequence with its homologs in tobacco, tomato and Arabidopsis. KNOX I and KNOX II subdomain, ELK domain, homeobox domain are marked. Amino acids in black shading and grey shading indicate identical or similar amino acids among the proteins.

(B) An unrooted phylogenetic tree of KNOX proteins in tobacco, tomato and Arabidopsis. The scale

indicates branch lengths. Accession numbers are as follows: TKN1 (Solyc04g077210.2), TKN2 (Solyc02g081120.2), NTH20 (BAA76904), BP (At4g08150), STM (At1g62360), NTH22 (BAA76905), NTH201 (BAF95776), KNAT2 (At1g70510), KNAT6 (At1g70510), KNAT5 (At4g32040), KNAT7 (At1g62990), KNAT4 (At5g11060), and KNAT3 (At5g25220), ATH1 (At4g32980).

(C) Tissue-specific expression of *NtBPL* in wild-type plants.

Supplementary Fig. S4. Developmental-specific expression of *NtSVP* and *NtBPL* in pedicels of wild-type plants.

Supplementary Fig. S5. Phenotypes of the *NtBPL* transgenic plants.

(A) The whole plant morphology of wild-type and *NtBPL* transgenic plants at the reproductive stage.

(B) Distribution of the angles between the pedicels and the inflorescences in WT, *NtBPL*-OE and *NtBPL*-RNAi plants. N= 10 X 5, 50 pedicels from five plants were measured as a replicate, and three replications were performed. Values are means ± SE.

(C) Longitudinal sections of the pedicels of WT and *NtBPL* transgenic plants at anthesis stage. Asterisks pointed to cortex cells. Bars = 100 μm.

(D) Cell numbers in the longitudinal cortex file of pedicels in WT and *NtBPL* transgenic plants at anthesis (N= 3 X 5, 15 sections from five individual plants per genotype were used). Values are means ± SE.

(E) qRT-PCR detection of *NtSVP* expression in pedicels of WT and *NtBPL* transgenic plants. *NtACTIN9* was used as an internal control.

Supplementary Fig. S6. The promoter sequence of *NtBPL*. The CArG-box and initiation codon were highlighted by black boxes.

Supplementary Fig. S7. Confirmation of microarray data. Four differently expressed genes *NtGRAS1*, *NtEREBP5*, *NtWRKY3*, and *NtSBP2* were randomly selected for qRT-PCR detection compared with those of microarray results. *NtACTIN9* was used as an internal control.

Supplementary Fig. S8. GO enrichment analysis of differentially expressed genes in *NtBPL*-RNAi pedicels and their MapMan classification.

(A) Gene ontology analysis of 427 down-regulated genes in *NtBPL*-RNAi pedicels compared with those in the wild type. Colors indicate significance level of each over-represented terms (FDR ≤ 0.01).

(B) Gene ontology analysis of 230 up-regulated genes in *NtBPL*-RNAi pedicels compared with those in the wild type plant. Colors indicate significance level of each over-represented terms (FDR ≤ 0.01).

(C) MapMan classification of differentially expressed genes in *NtBPL*-RNAi pedicels.

Supplementary Table S1. Hormone related genes that are differentially expressed due to down-regulation of *NtBPL*.

Supplementary Table S2. Primers used in this work.

Supplementary dataset 1. List of differentially expressed genes between the wild type and *NtBPL*-RNAi pedicels.

Fig. S1

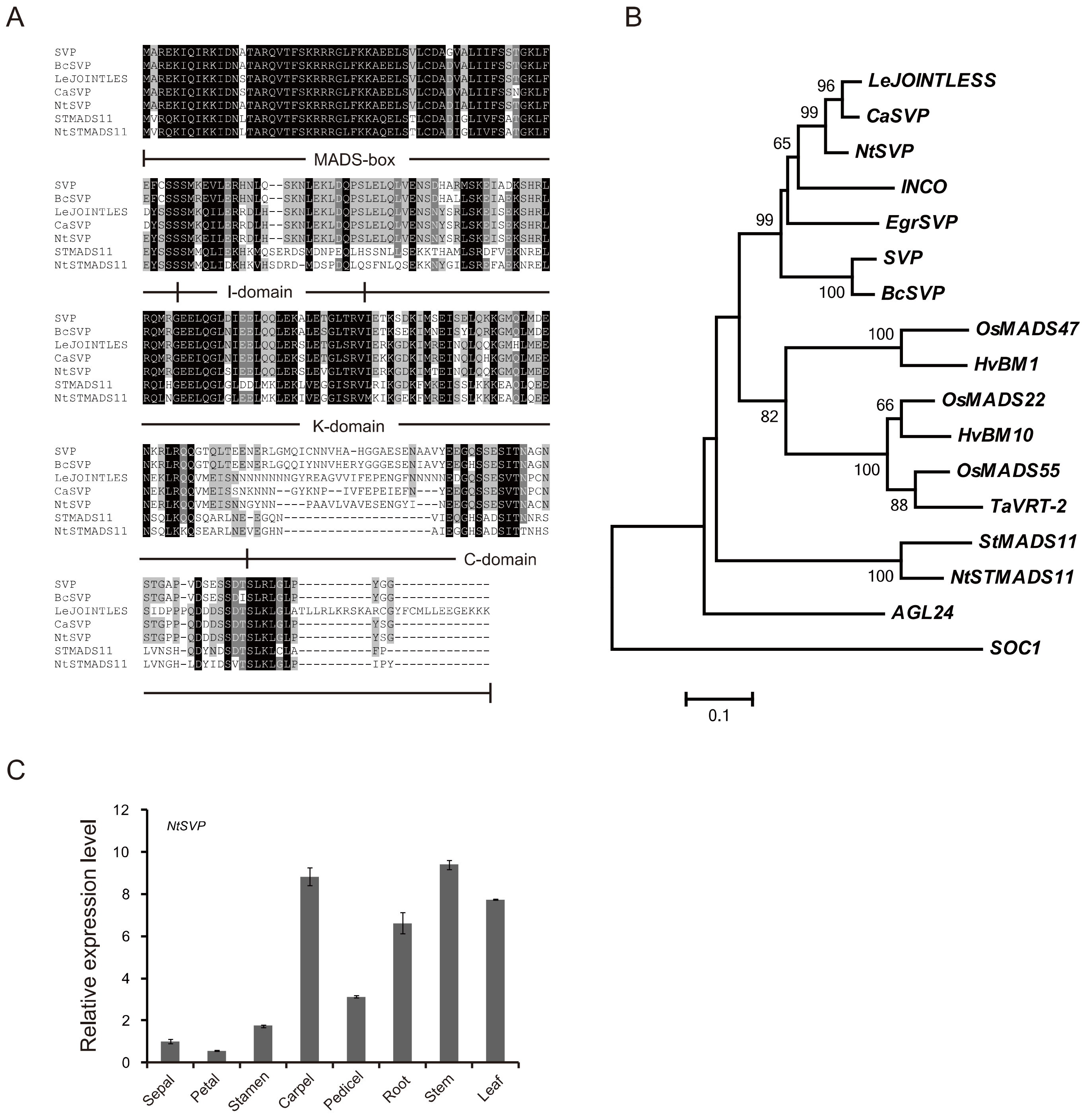

A
SVP
BcSVP
LeJOINTLES
CaSVP
NtSVP
STMADS11
NtSTMADS11
MADS-box
I-domain
K-domain
C-domain
B
LeJOINTLESS
CaSVP
NtSVP
INCO
EgrSVP
SVP
BcSVP
OsMADS47
HvBM1
OsMADS22
HvBM10
OsMADS55
TaVRT-2
StMADS11
NtSTMADS11
AGL24
SOC1
96
99
65
99
100
100
66
82
100
88
100
0.1
C
NtSVP
Relative expression level
12
10
8
6
4
2
0
Sepal
Petal
Stamen
Carpel
Pedicel
Root
Stem
Leaf

Fig. S2

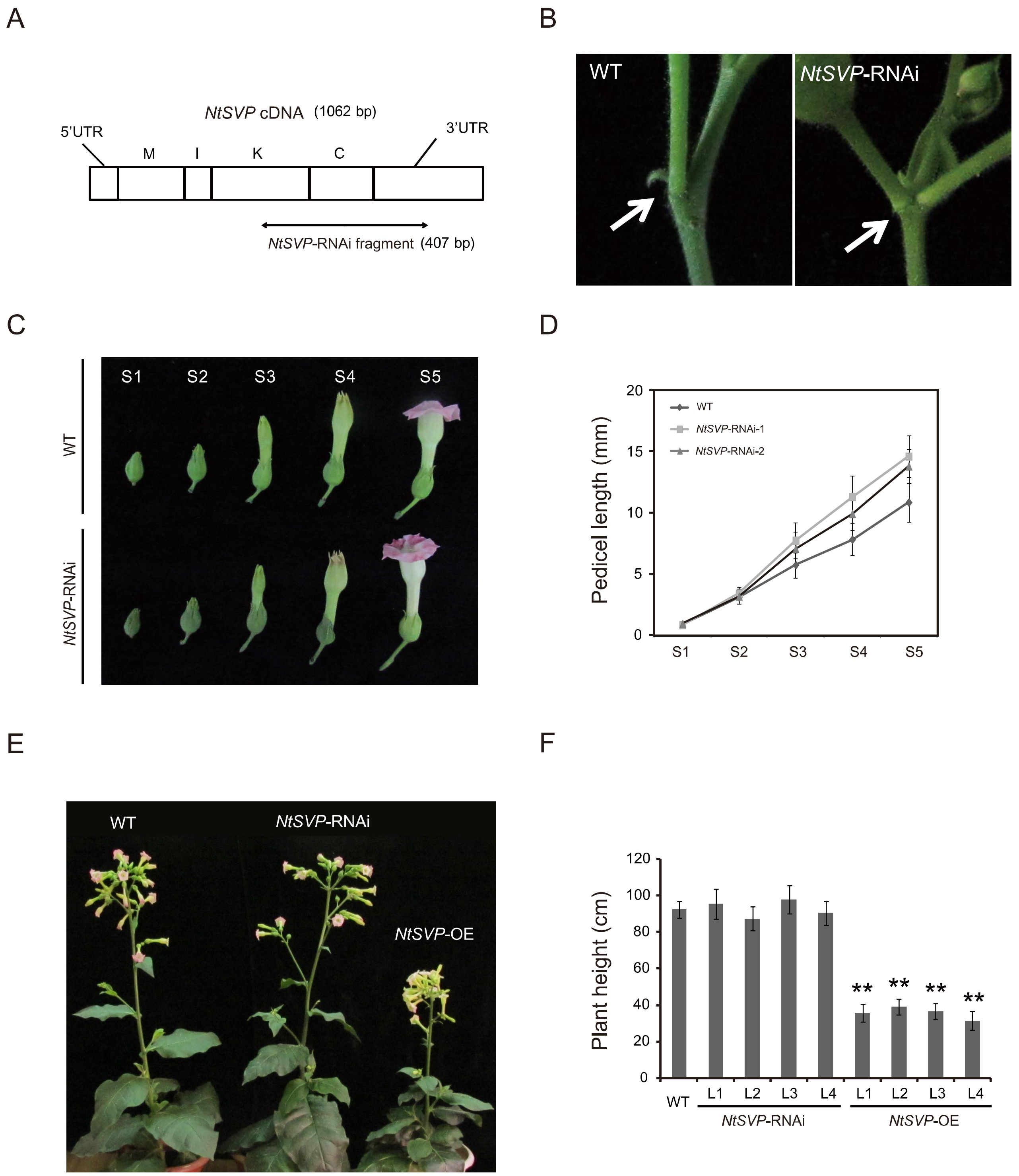
A
B
NtSVP cDNA (1062 bp)
5'UTR
3'UTR
M
I
K
C
NtSVP-RNAi fragment (407 bp)
WT
NtSVP-RNAi
C
D
S1
S2
S3
S4
S5
WT
NtSVP-RNAi
NtSVP-RNAi-1
NtSVP-RNAi-2
Pedicel length (mm)
20
15
10
5
0
E
F
WT
NtSVP-RNAi
NtSVP-OE
Plant height (cm)
120
100
80
60
40
20
0
WT
L1
L2
L3
L4
**
NtSVP-RNAi
NtSVP-OE

Fig. S3

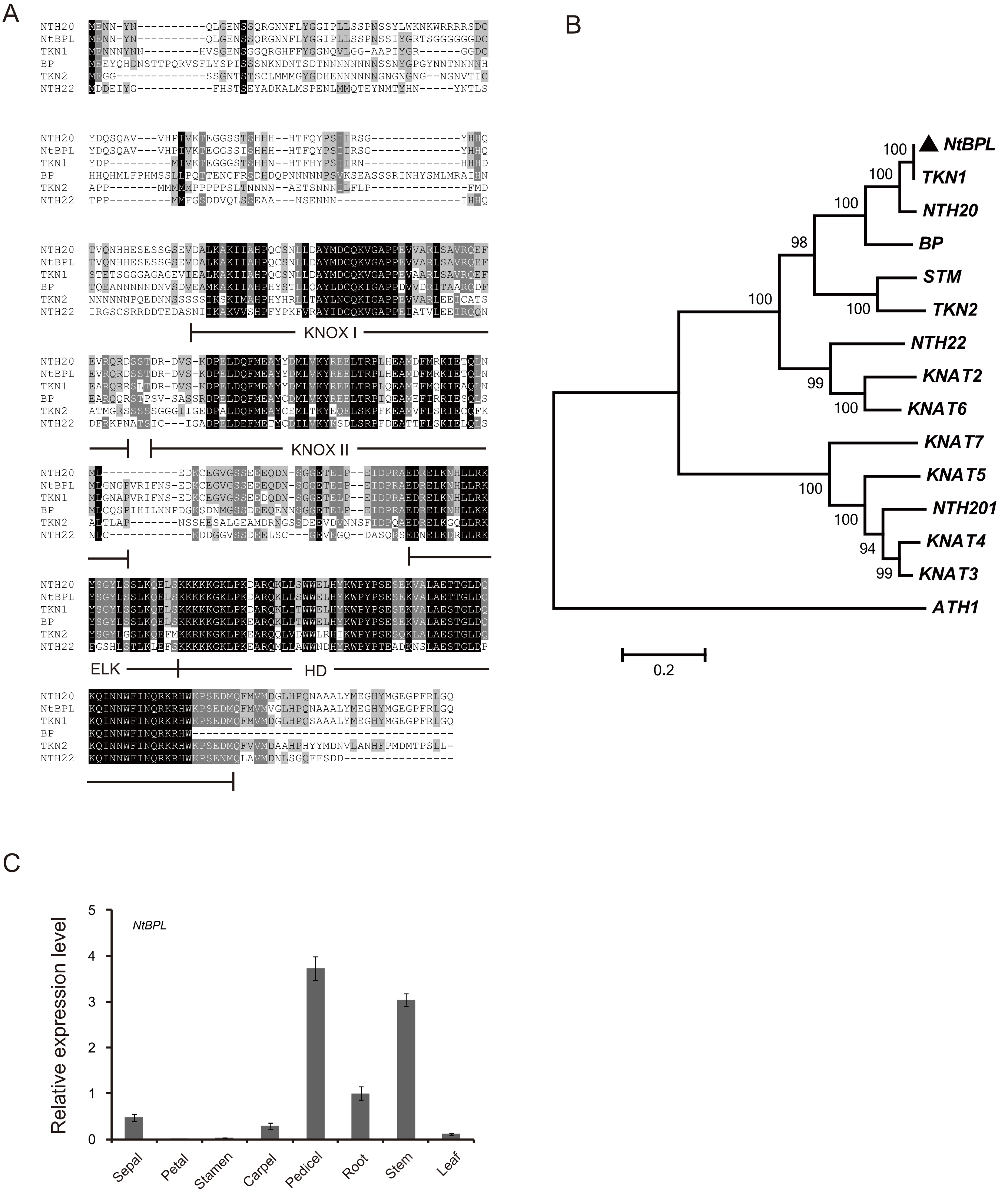
A
NTH20
NtBPL
TKN1
BP
TKN2
NTH22
KNOX I
KNOX II
ELK
HD
B
NtBPL
TKN1
NTH20
BP
STM
TKN2
NTH22
KNAT2
KNAT6
KNAT7
KNAT5
NTH201
KNAT4
KNAT3
ATH1
100
98
99
94
0.2
C
NtBPL
Relative expression level
0
1
2
3
4
5
Sepal
Petal
Stamen
Carpel
Pedicel
Root
Stem
Leaf

Fig. S4

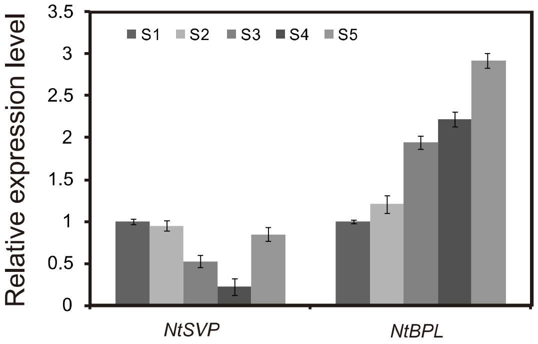

Relative expression level
3.5
3
2.5
2
1.5
1
0.5
0
S1
S2
S3
S4
S5
NtSVP
NtBPL

Fig. S5

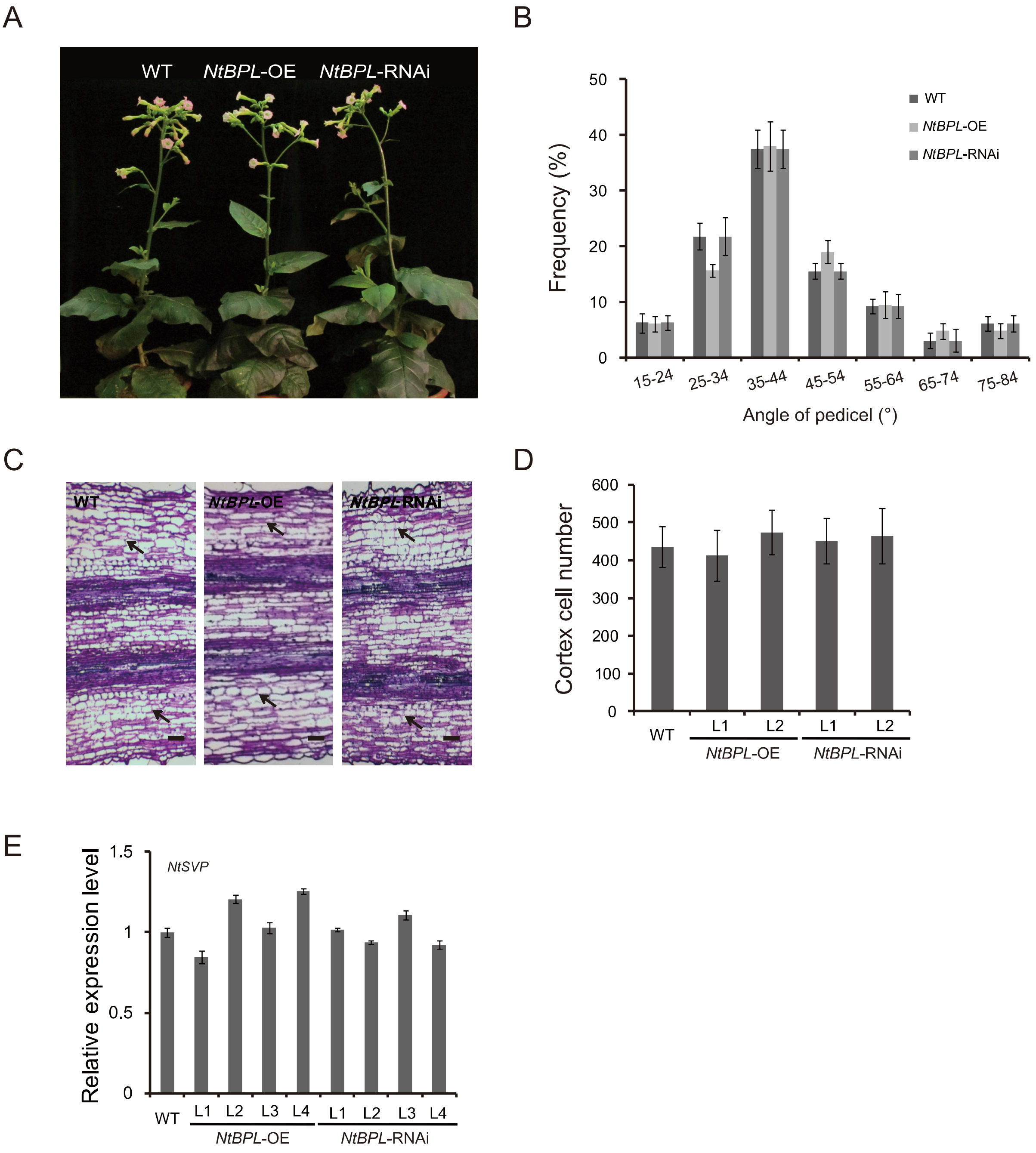
A
WT
NtBPL-OE
NtBPL-RNAi
B
Frequency (%)
50
40
30
20
10
0
WT
NtBPL-OE
NtBPL-RNAi
15-24
25-34
35-44
45-54
55-64
65-74
75-84
Angle of pedicel (°)
C
WT
NtBPL-OE
NtBPL-RNAi
D
Cortex cell number
600
500
400
300
200
100
0
WT
L1
L2
L1
L2
NtBPL-OE
NtBPL-RNAi
E
Relative expression level
1.5
1
0.5
0
NtSVP
WT
L1
L2
L3
L4
L1
L2
L3
L4
NtBPL-OE
NtBPL-RNAi

Fig. S6

```
GGCCTACTAA TAGGGGGCGA TTGAGCTGCC CTTAGCTTCG CTTCTTCTAT CAATTCCTTG
CCATCATCCT CTTTGATAAT GTAAACAATT GAACCAATTT ATATAACTCA ATTTTTCTAT
CAATTATAAG AAACTAACTT TTCCAAGGTA GTGAAATAAA TAATCAATAG AAAAAGAAAG
ACCTCACAAA AGGAAAAAGA AAATGAAAAG GGCTTCCCAT GGAAGTAATA GGAAAAGCAA
AAGAATACAA AAAGAAAGAG AAAGTTAAAA GATCTAACTA ACTAACTCTA TATATATACA
TATATATATA TTGAATAAAA TAATTATGCT TTCTTTATAA TTGAGTGGTT TTCTCTGCAT
AGGATTATAG GGGGTATAGT TAAACTAAAG ACTAATATTT TCCAATACAT TTCCTTATTC
TTCAACGTAA GAACGTCTTG TCATCTACGA AGCCGCTACC AGGAGCGGCC ACTAGCTTAC
CCCTTCCCTT CCTTTCCTTT CCTTTTTTCT TTGTTCACAG CCACTATCTT CTATGTTGTC
AAGATAAGTT CATTCCAAAA CTTCTTCAGT ACAACTTCAT CTCCTTGGCC TCCTTAACCA
GACAACACTA GTAAAAATAT CCAATTATTA TTATTTTTTG TGTGTGTTTC TTTTACCAAA
TAAAGAAGAG ATCTCGAAGA GTTAAATGAA CTTTCCCCCT ATAGTTGTGT TAGTTTGACG
AATAATAAGT ATTCTAGACT TTGTTTAGGG TTTCATCTAG TTCTCAACTG TTTATCCACA
TCTTTGATTT AGGGTTGCCG ATCTTCATAT ATTTTTTTTA CCATATCTTG TTAGGGTAAT
TATCTAGAAA GGGCAGCTTG GCGTAATCAT GGTCATAGCT GTTTCCTGTG TGAATTGTTA
TCCGCTCACA ATTCCACACA ACATACGAGC CGGAAGCATA AAGTGTAAAG CCTGGGGTGC
TAATGAGTGA GCTAACTCAC ATTATTATGG AGAATAATTA
```

CArG-box: **CAATTATAAG**

Initiation Codon: **ATG**

Fig. S7

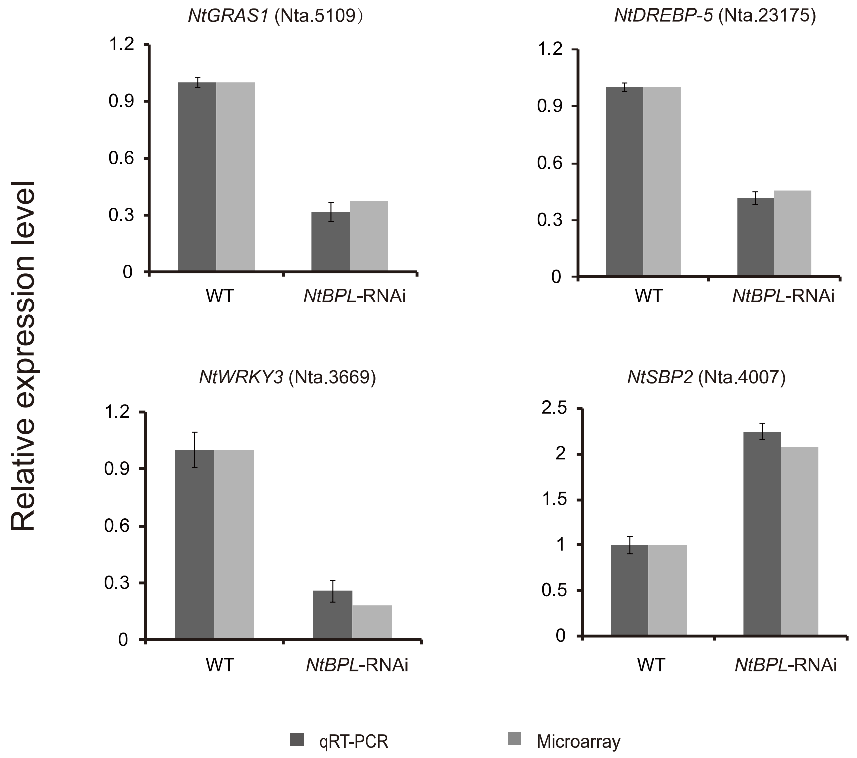
NtGRAS1 (Nta.5109）
NtDREBP-5 (Nta.23175)
NtWRKY3 (Nta.3669)
NtSBP2 (Nta.4007)
Relative expression level
1.2
0.9
0.6
0.3
0
2.5
2
1.5
1
0.5
WT
NtBPL-RNAi
qRT-PCR
Microarray

Fig. S8

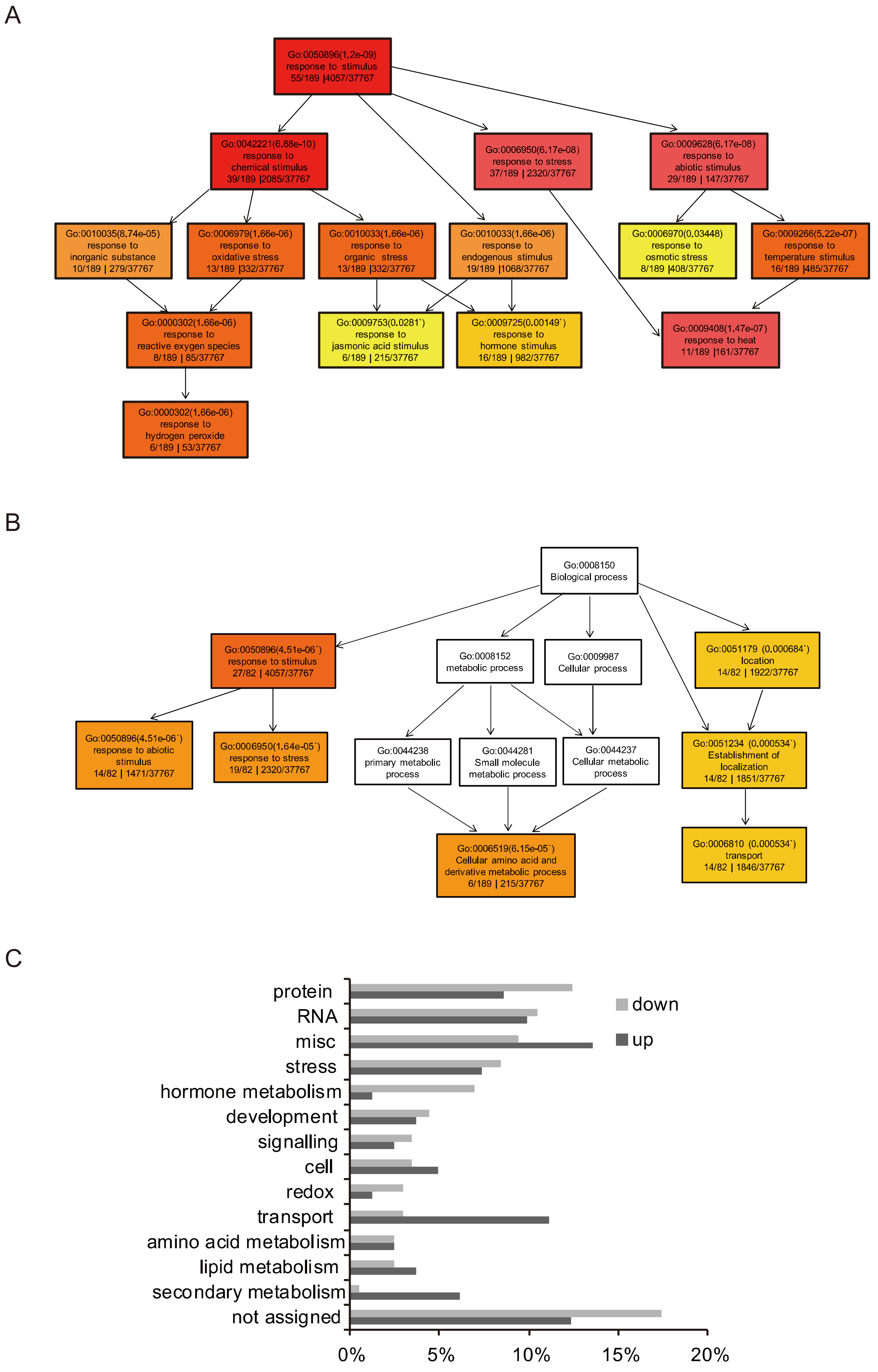
A
Go:0050896(1.2e-09)
response to stimulus
55/189 |4057/37767
Go:0042221(6.88e-10)
response to
chemical stimulus
39/189 |2085/37767
Go:0006950(6.17e-08)
response to stress
37/189 | 2320/37767
Go:0009628(6.17e-08)
response to
abiotic stimulus
29/189 | 147/37767
Go:0010035(8.74e-05)
response to
inorganic substance
10/189 | 279/37767
Go:0006979(1.66e-06)
response to
oxidative stress
13/189 |332/37767
Go:0010033(1.66e-06)
response to
organic stress
13/189 |332/37767
Go:0010033(1.66e-06)
response to
endogenous stimulus
19/189 |1068/37767
Go:0006970(0.03448)
response to
osmotic stress
8/189 |408/37767
Go:0009266(5.22e-07)
response to
temperature stimulus
16/189 |485/37767
Go:0000302(1.66e-06)
response to
reactive exygen species
8/189 | 85/37767
Go:0009753(0.0281`)
response to
jasmonic acid stimulus
6/189 | 215/37767
Go:0009725(0.00149`)
response to
hormone stimulus
16/189 | 982/37767
Go:0009408(1.47e-07)
response to heat
11/189 |161/37767
Go:0000302(1.66e-06)
response to
hydrogen peroxide
6/189 | 53/37767
B
Go:0008150
Biological process
Go:0050896(4.51e-06`)
response to stimulus
27/82 | 4057/37767
Go:0008152
metabolic process
Go:0009987
Cellular process
Go:0051179 (0.000684`)
location
14/82 | 1922/37767
Go:0050896(4.51e-06`)
response to abiotic
stimulus
14/82 | 1471/37767
Go:0006950(1.64e-05`)
response to stress
19/82 | 2320/37767
Go:0044238
primary metabolic
process
Go:0044281
Small molecule
metabolic process
Go:0044237
Cellular metabolic
process
Go:0051234 (0.000534`)
Establishment of
localization
14/82 | 1851/37767
Go:0006519(6.15e-05`)
Cellular amino acid and
derivative metabolic process
6/189 | 215/37767
Go:0006810 (0.000534`)
transport
14/82 | 1846/37767
C
protein
RNA
misc
stress
hormone metabolism
development
signalling
cell
redox
transport
amino acid metabolism
lipid metabolism
secondary metabolism
not assigned
down
up
0%
5%
10%
15%
20%

Supplementary Table S1. Hormone related genes that are differentially expressed due to down-regulation of *NtBPL* .

	Arabidopsis homologs	Description	Foldchange	UniGene ID
Up regulated				
auxin.induced-regulated-responsive-activated	at5g54510	GH3.6, DFL1 | DFL1 (DWARF IN LIGHT 1)	2.3	Nta.23121
gibberelin.induced-regulated-responsive-activated	at3g02885	GASA5	6.01	Nta.8147
Down regulated				
auxin.induced-regulated-responsive-activated	at2g45210	Auxin-responsive protein-related	0.24	Nta.3096
auxin.induced-regulated-responsive-activated	at5g35735	Auxin-responsive family protein	0.297	Nta.8872
auxin.induced-regulated-responsive-activated	at4g27450	Unknown protein	0.42	Nta.801
brassinosteroid.synthesis-degradation.BRs.metabolic regulation	at2g26710	BAS1, CYP734A1, CYP72B1	0.48	Nta.15867
ethylene.synthesis-degradation.1-aminocyclopropane-1-carboxylate synthase	at5g65800	ACS5, CIN5, ETO2, ATACS5	0.35	Nta.14789
ethylene.synthesis-degradation.1-aminocyclopropane-1-carboxylate oxidase	at2g19590	ACO1	0.15	Nta.12328
gibberelin.synthesis-degradation.copalyl diphosphate synthase	at4g02780	GA1, ABC33, ATCPS1	0.22	Nta.4073
gibberelin.synthesis-degradation.ent-kaurene synthase	at1g79460	GA2, KS, ATKS	0.38	Nta.23734
gibberelin.synthesis-degradation.ent-kaurenoic acid hydroxylase/oxygenase	at1g05160	CYP88A3, ATKAO1, KAO1	0.47	Nta.4075
gibberelin.synthesis-degradation.GA3 oxidase	at1g15550	GA4, ATGA3OX1, GA3OX1	0.33	Nta.3870
gibberelin.synthesis-degradation.GA3 oxidase	at1g80340	GA4H, ATGA3OX2, GA3OX2	0.18	Nta.9957
gibberelin.induced-regulated-responsive-activated	at2g14900	GASA7	0.23	Nta.3268

Supplementary Table S2. Primers used in this work

	Sequence (5'-3')
**For transgenic plants**	
NtSVP RNAi-F	GTCATAGAGA GAAAG
NtSVP RNAi-R	GCCAAACATG AATCTGA
NtSVP OE-F	CGATGAGCTCATGGCTAGAGAGAAAATT
NtSVP OE-R	CGTAGGTACCTCAACCTGAGTAGGGCAG
**For RACE**	
NtSVP gstWR	TTTCTCTCTATGACGCGGGTCAATCCAG
NtSVP -F1	TCCAATAATGGATACAACAACCCAG
NtSVP -F2	GCAGCAGTATTAGTAGCAGTTGAAT
Gene Racer 5' primer	CGACTGGAGCACGAGGACACTGA
**For TAIL-PCR**	
NtBPL -SP1	CCGCTTGTTCTTCCATAAATAG
NtBPL -SP2	TATGGGATGTACCACTGCTTG
NtBPL -SP3	GATGAGTTCTCCCCCAGTTGAT
**For real-time PCR**	
NtACTIN9 -F	CTATTCTCCGCTTTGGACTTGGCA
NtACTIN9 -F	AGGACCTCAGGACAACGGAAACG
NtSVP -F	TGTGATGCTGATGTTGCTCTC
NtSTMADS11 -F	AGAGCTGCAAGGACTTGGCTT
NtSTMADS11 -R	TGATGGAGTCTGCGGAATGTC
NTH20- Fw	TACAATCAACTGGGGGAGAAC
NTH20 -Rv	CTACGAATAATCGAAGGGTA
NTH22 -F	AGTTTGGGAGTCACCTAAGTAC
NTH22 -R	AGCTAACTGCATATTCTCTGAC
NtSVP -R	GTTCTTCTCCCCTCATTTGTC
NtBPL -F	AGCAGTGGTACATCCCATAGT
NtBPL -R	CTCACTTCAAACTCTTGCCGT
NtCPS- F	TCAAAGAGAAGATGGATCTTGGG
NtCPS- R	AGGTGAAATCTGATCCTTGTTATC
NtKS -F	GTCATGGGCCAAGAATGGTG
NtKS-R	CGCTGAAAATATGATGCCCAC
NtKAO -F	TGGGGATGGTATAACCATTAATCAT
NtKAO -R	TGAATCTTCTGGTCATATTACAATG
NtGA3ox1 -F	GCCTTTTTTCGCTTCCAATGC
NtGA3ox1 -R	GCTTTTCCATTTCCCTTTCGT
NtGA3ox2 -F	GAGGAAGCTGGAAAACGACTT
NtGA3ox2 -R	AGCATCAGCCACATCAGTCTT
NtAS1- F	AGAGGAGCAGCGTCTTGTTAT
NtAS1- R	GACACTCCGCTCTTTCACAAT
NtAS2- F	TCTACCGTGGCTGATCCATAC
NtAS2- R	AGAAACCTCGAACCATCCTTG
NtER- F	AAACAGCTGGAACTGCTGAAC
NtER- R	CGCACATCAAAATACCACAGG
NtCRM- F	CTGTTCAGGCTAATGTGGTAT
NtCRM -R	GGGATGATGGACAAACAAGAG
NtYUCC5 -F	AGTTTCTCAGATAACGATTTCTTTGC
NtYUCC5 -R	GTCTCTTTGTAGGGTACTCAGGGTAG
**For yeast one hybrid**	
NtSVP -F	aagcagtggtatcaacgcagagtggccattatggcccATGGCTAGAGAGAAAATTCAGATCA
NtSVP -R	AGATCTCCGGCTCCGCCGGCTGTACACCTGAGTAGGGCAGCCCCAACTTG
CArG-box	GAGCTCGCAATTAAAAGACGCTATTAAAAGACGCTATTAAAAGACCTCGA
CArG-box(m)	GAGCTCGCAAGGAAAAGACGCTATTAAAAGACGCTATTAAAAGACCTCG
**Probes for EMSA**	
mu-pro*NtBPL* (+)	actcaatttttctatGGATGCATCCaaactaacttttcca
mu-pro*NtBPL* (-)	TGGAAAAGTTAGTTTGGATGCATCCATAGAAAAATTGAGT
wt-pro*NtBPL* (+)	ACTCAATTTT TCTATCAATT ATAAGAAACT AACTTTTCCA
wt-pro*NtBPL* (-)	TGGAAAAGTTAGTTTCTTATAATTGATAGAAAAATTGAGT
BamHI-*NtSVP* Fw	CGAggatccATGGCTAGAGAGAAAATTCAG
XholI-*NtSVP* Rv	CGCctcgagACCTGAGTAGGGCAGCCC
**For dual-luciferase assay**	
Sal I-pro*NtBPL* -Fw	cacGTCGACgcttcgcttcttctatcaattc
Bam HI-pro*NtBPL* -Rv	cgcGGATCCTAATTACCCTAACAAGATATGGT
m1CArG-R	TACCTTGGAAAAGTTAGTTTATTATAATTTATAGAAAAATTGAG
m1CArG-F	ACTCAATTTTTCTATAAATTATAATAAACTAACTTTTCCAAGGT
m2CArG-R	ACCTTGGAAAAGTTAGTTTATTAGCATTTATAGAAAAATTGAGT
m2CArG-F	ACTCAATTTTTCTATAAATGCTAATAAACTAACTTTTCCAAGGT
